# 

**Published:** 1900-04

**Authors:** 


### BOOKS RECEIVED.

Venereal Diseases, their Complications and Sequelæ. By Edward L. Keyes, A. M., M. D., and Charles H. Chetwood, M. D. One volume 8vo. Extra muslin, $2.75, net. New York: William Wood & Co. 1900.

A Pocket Medical Dictionary, giving the pronunciation and definition of the principal words used in medicine and the collateral sciences, including very complete tables of clinical eponymic terms; of the arteries, muscles, nerves, bacteria, bacilli, micrococci, spirilla and thermometric scales; and a dose-list of drugs and their preparations in both the English and metric systems of weights and measures. By George M. Gould, A. M., M. D. Fourth edition, revised and enlarged, containing 30,000 words, Philadelphia: P. Blakiston's Son & Co. 1900. Price, $1.00.

Diseases of the Nose and Throat. By J. Price Brown, M. B., L.R.C.P.E., Member of the College of Physicians and Surgeons of Ontario; Laryngologist to the Toronto Western Hospital; Laryngologist to the Protestant Orphans' Home; Fellow of the American Laryngological, Rhinological, and Otological Society; Member of the British Medical Association, the Pan-American Medical Congress, the Canadian Medical Association, the Ontario Medical Association, etc., etc. Illustrated with 159 engravings, including six full-page color-plates and nine color-cuts in the text, many of them original. 6¼ x 9¼ inches. Pages xvi.–470. Extra cloth, $3.50, net. Philadelphia: The F. A. Davis Co., Publishers. 1900.

On Diabetes Mellitus and Glycosuria. By Emil Kleen, Ph. D., M. D. Octavo, 313 pages. Price, cloth, $2 50, net. Philadelphia: Blakiston's Son. & Co., 1012 Walnut street. 1900.

The Irrigation Treatment of Gonorrhea. Its Local Complications and Sequelæ. By Ferd. C. Valentine, M. D One volume, 8vo, profusely illustrated. Muslin, $2.00, net. New York: William Wood & Company. 1900.

Scattered Leaves from a Physician's Diary. A series of satirical sketches from real life, reflecting more or less upon the men who control it, by Albert Abrams, A. M., M. D. (Heidelberg), F. R. M. S, San Francisco, author of "The Antiseptic Club," etc.; pp. 60, with frontispiece. Price, 50 cents. St. Louis, Mo: Fortnightly Press Co., Publishers. 1900.

The International Text-Book of Surgery. By American and British Authors. Edited by J. Collins Warren, M. D., LL. D., Professor of Surgery in Harvard Medical School; Surgeon to the Massachusetts General Hospital; and A. Pearce Gould, M. S, F. R. C. S., Surgeon to Middlesex Hospital, and Teacher of Operative Surgery at Middlesex Hospital Medical School; Member of the Court of Examiners of the Royal College of Surgeons, England. Volume II. Regional Surgery. With 471 illustrations in the text, and eight full-page plates in colors. Philadelphia: W. B. Saunders. 1900. Price, $5.00.

Surgical Pathology and Therapeutics. By John Collins Warren, M. D., LL. D, Professor of Surgery in Harvard University; Surgeon to the Massachusetts General Hospital. Illustrated. Second edition, with an appendix, containing an enumeration of the Scientific Aids to Surgical Diagnosis, together with a series of Sections on Regional Bacteriology. Philadelphia: W. B. Saunders. 1900.

